# Specific Changes of Serum Proteins in Parkinson's Disease Patients

**DOI:** 10.1371/journal.pone.0095684

**Published:** 2014-04-25

**Authors:** Wenwen Lu, Xinhua Wan, Bin Liu, Xianfang Rong, Lei Zhu, Pingping Li, Jiang Li, Ling Wang, Liying Cui, Xiaoliang Wang

**Affiliations:** 1 State Key Laboratory of Bioactive Substances and Functions of Natural Medicines, Department of Pharmacology, Institute of Materia Medica, Chinese Academy of Medical Sciences & Peking Union Medical College, Beijing, China; 2 Department of Neurology, Peking Union Medical College Hospital, Chinese Academy of Medical Sciences & Peking Union Medical College, Beijing, China; CHA University, Korea, Republic of

## Abstract

The aim of this study is to identify and validate protein change in the serum from PD patients. We used serum samples from 21 PD patients and 20 age-matched normal people as control to conduct a comparative proteomic study. We performed 2-DE and analyzed the differentially expressed protein spots by LC-MS/MS. In PD group 13 spots were shown to be differentially expressed compared to control group. They were identified as 6 proteins. Among these, 3 proteins were confirmed by Western blot analysis. It showed that the frequency of fibrinogen γ-chain (FGG) appeared 70% in PD, which could not be detected in control group. The protein of inter-alpha-trypsin inhibitor heavy chain H4 (ITI-H4) was found to exist two forms in serum. The full size (120 kDa) of the protein was increased and the fragmented ITI-H4 (35 kDa) was decreased in PD group. The ratio of full size ITI-H4 to fragmented ITI-H4 in PD patients was 3.85±0.29-fold higher than in control group. Furthermore, fragmented Apo A-IV (∼26 kDa) was mainly detected in control group, while it was rare to be found in PD group. Above findings might be useful for diagnosis of PD. When the expressions of FGG and 120 kDa ITI-H4 are increase, as well as ∼26 kDa Apo A-IV disappear would provide strong evidence for PD.

## Introduction

Parkinson's disease (PD) is the second most common neurodegenerative disease after Alzheimer's disease (AD), characterized by progressive and profound loss of neuromelanin containing dopaminergic neurons in the substantia nigra pars compacta (SNpc) with presence of eosinophillic, intracytoplasmic, proteinaceous inclusions termed as Lewy bodies (LB) and dystrophic Lewy neurites in surviving neurons [1]. Clinical features of PD include motor impairments involving resting tremor, bradykinesia and rigidity along with non-motoric symptoms like autonomic, cognitive and psychiatric problems. At early stages, the accurate clinical diagnosis remains challenging [2], [3]. A clinical validation for PD can be time-consuming, with multiple tests including motor, olfactory, visual, and psychological assessments, imaging (MRI, PET), as well as biochemical testing of cerebrospinal fluid (CSF), lung, liver, heart, and lymphocytes [4]–[6]. By the time symptoms of PD are manifest, substantial neurodegeneration has already occurred. Therefore, an early diagnostic test that accurately detects PD is essential to evaluate and implement early intervention strategies. In addition, although mitochondrial, proteasomal dysfunction and oxidative stress are widely recognized as major contributors, the mechanisms underlying the development of PD remain unknown [7], [8]. The discovery of key proteins related to the development and progression of PD would not only shed greater light on PD pathogenesis, but would also provide new targets for drug discovery.

Proteomic analysis has been used to understand the molecular mechanism of PD and to develop biomarkers for its early diagnosis [9]–[11]. Moreover, it can resolve distinct isoforms of protein and identify post-translational modifications which might be functionally important [12]. Several studies have investigated biochemical biomarkers for this disease in various tissues including CSF and blood [13]–[15]. Although many studies have been done in biomarker research for PD, still no validated, inexpensive, and simple markers are available. Since that lumbar puncture is a relatively invasive procedure and obtaining CSF on large numbers of elderly infirm individuals in the community is challenging, blood is easy to obtain and as ∼500 ml of CSF are absorbed into the blood every day, serum may offer a rich source of disease biomarkers. Furthermore damage to the blood-brain barrier [16], a known event in this disease, may enhance movement of proteins between brain and blood, in either direction. However, serum contains a small group of high-abundance proteins, including albumin, immunoglobulins, transferrin and macroglobulin, which constitutes about 85% of the total serum protein, may interfere with the identification of low-abundance proteins. Thus, optimal proteomic analysis of human serum requires depletion of high-abundance proteins to facilitate observation of low-abundance proteins. With removal of the high-abundance proteins, the remaining proteins can be identified over a relatively high dynamic range. In the present study, we performed two-dimensional gel electrophoresis (2-DE)-based proteomic analysis of serum samples from 21 PD patients and 20 same age range normal volunteers. The differentially expressed proteins from the PD patients were identified by liquid chromatography in combination with tandem mass spectrometry (LC-MS/MS). Our study provided a group of interesting candidate molecules which might help to develop potential diagnostic markers of PD.

## Results

### Depletion of the highly abundant serum proteins

**Figure 1:**
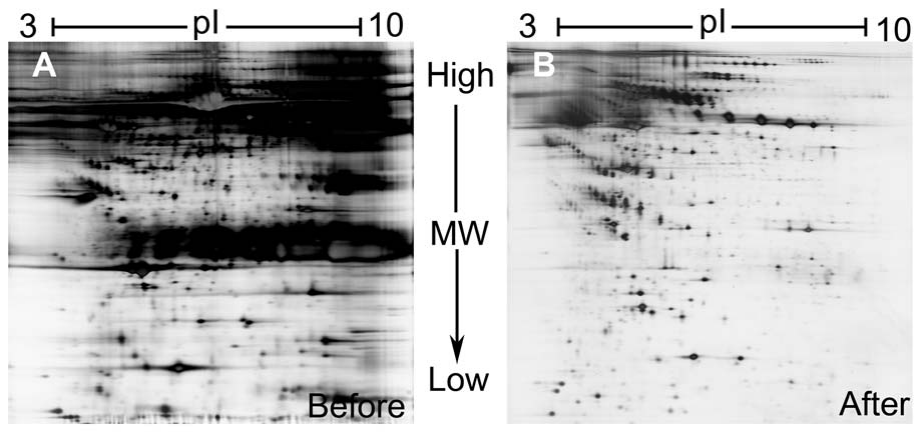
Serum depletion. It was clear from 2-DE of whole serum and depleted serum proteins that many more proteins were visible in the depleted sample (B) than were in the whole serum sample (A) when both gels were loaded with the same overall amount of serum proteins.

In this study, a comparative proteomic research was conducted using serum samples from PD patients and normal men as control. In order to remove the most abundant proteins from the serum, an immune affinity column (MARS), which was an easy-to-use method for simultaneously removing albumin, IgG, antitrypsin, IgA, haptoglobin, transferrin, fibrinogen, alpha2-macroglobin, alpha1-acid glycoprotein, IgM, apolipoprotein AI, apolipoprotein AII, complement C3 and transthyretin in serum with relatively high specificity, was applied to collect flow-through factions. Alterations in protein expression associated with PD were then investigated by applying 2-DE-based proteomic analysis on the depleted serum samples. Figure 1 shows the representative 2-D gels of whole serum and the fully depleted serum. Clearly, the depleted serum allows visualization of many more low abundant proteins.

### Protein spots changed in the serum of PD patients versus controls

**Figure 2:**
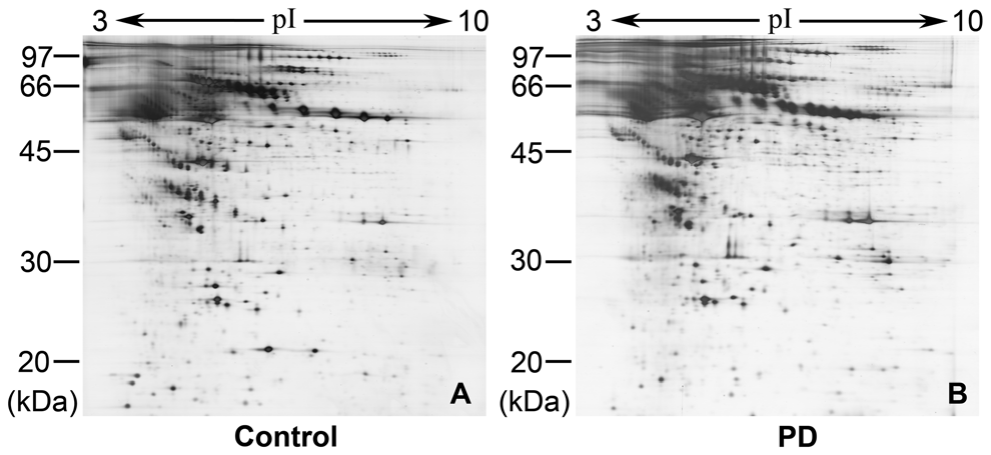
Representative silver-stained 2-DE gels of serum proteins from controls (A) and PD patients (B). The most dominant proteins in human sera derived from controls and PD patients were depleted by an immunoaffinity column (MARS) and then separated by 2-DE. Molecular weight is indicated on the left side in kDa.

Total proteins of depleted serum samples taken from 21 PD patients and 20 controls were separated by reproducible high-resolution 2-DE respectively to identify differentially expressed proteins. In another word, PD patient group contained twenty one individual 2-DE gels, and control group contained twenty individual 2-DE gels. The overall protein expression patterns in the gels were very similar between PD and control groups, and the representative 2-DE separation profiles were shown in Figure 2. Approximate 800 spots were detected in each silver-stained gel by the image software. The expression level was determined by examining the ratio of the relative spot volume of a protein in the gel. The ratios of normalized spot volume between PD and control groups were calculated, and the spots showing more than two fold difference with statistical significance (*P*<0.05) were considered as changed.

**Figure 3:**
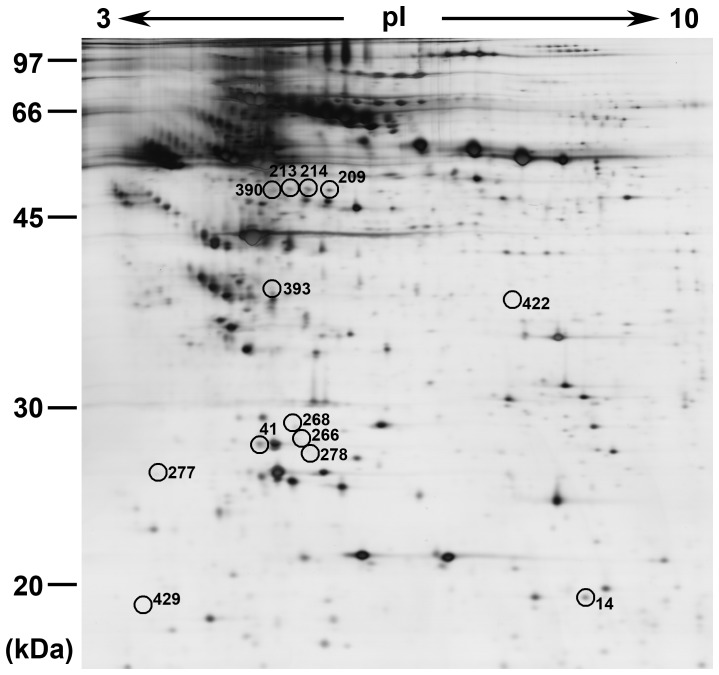
Distribution of differentially expressed protein spots. The samples were separated using IPG gel (pH 3–10, 18 cm) in the first phase and 12.5% SDS-PAGE; 120 µg of protein was used in each gel. The spots showing significant differences between PD patients and controls (see Table 1) were labeled in a 2-DE gel from PD group.

**Figure 4:**
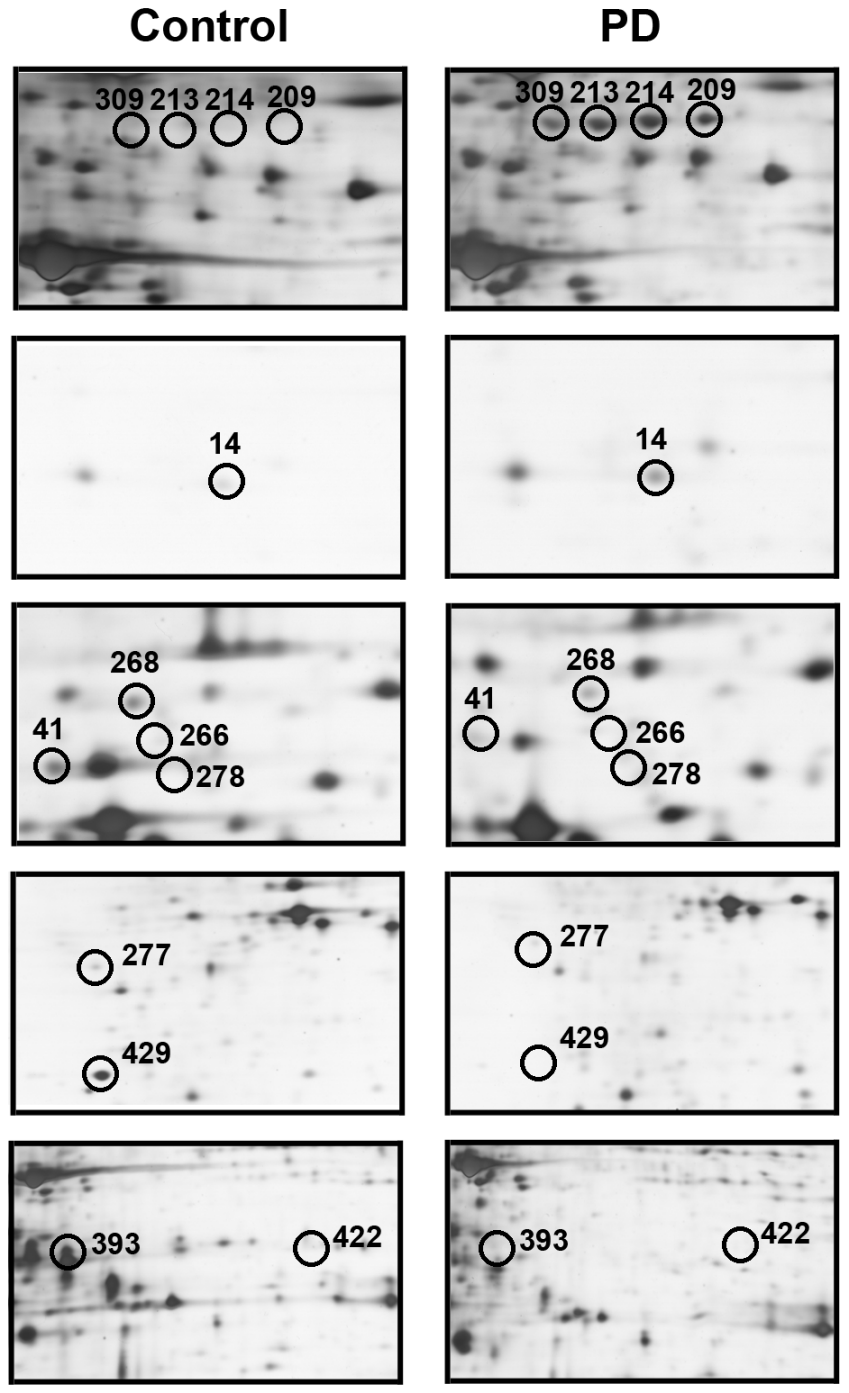
Expanded images of protein spots differentially expressed between PD patients and controls. The protein FGG (spot 390, 213, 214 and 209) and haptoglobin (spot 14) were increased in abundance in the serum from PD patients, relative to the levels in controls, whereas the levels of prothrombin (spot 429 and 277), inter-alpha-trypsin inhibitor heavy chain H4 (spot 422 and 393), apolipoprotein A-I (spot 278 and 41) and apolipoprotein A-IV (spot 268 and 266) were decreased in PD serum relative to the levels in controls.

Up to 13 protein spots were found differentially expressed in the serum from PD patients in comparison with controls (Figure 3). Among them, 3 protein spots (spots 14, 214 and 390) were up-regulated (their variation ratios were more than +2), 2 spots (209 and 213) presented only in PD patients(their variation ratios were +10000), 7 spots (277, 422, 278, 268, 393, 266, and 41) were down-regulated (their variation ratios were less than −2), and 1 spot (429) disappeared (its variation ratio was −10000). The quantitative changes in these proteins and the ratio of PD expression levels to control levels are shown in Table 1. Enlarged regions of the respective gels are shown in Figure 4.

**Table 1:** Variation ratios of the protein spots differentially expressed in the serum from PD patients versus controls.

Rank	Spot No.	*P*-value	*q*-value	Variation ratio	State change
1	429	0.0218	0.259	−10000	↓PD
2	209	0.0012	0.051	10000	↑PD
3	213	0.0013	0.045	10000	↑PD
4	277	0.0013	0.049	−97.83	↓PD
5	422	0.0003	0.030	−17.64	↓PD
6	214	0.0004	0.033	9.52	↑PD
7	390	0.0239	0.275	6.93	↑PD
8	278	0.0288	0.281	−4.15	↓PD
9	268	0.0082	0.173	−4.09	↓PD
10	393	0.0099	0.189	−3.78	↓PD
11	14	0.0408	0.293	2.28	↑PD
12	266	0.0349	0.282	−2.17	↓PD
13	41	0.0007	0.040	−2.09	↓PD

The serum proteome of the PD patients were compared with controls by ImageMaster 2D Platinum software. Spots with *P*-values<0.05 and >2-fold variation ratio between groups were considered as the differential spots. “Ratio = 10000” means the spot presented only in one group. *q*-values represent the FDR adjusted *P*-values.

To further analyze the differentially expressed protein spots and solve the problem of multiple hypotheses testing [17], the *P*-values were adjusted to *q*-values by the false discovery rate (FDR), and the cutoff of *q*-value was 0.05. As shown in Table 1, five out of thirteen spots (213, 277, 422, 214 and 41) also met the condition that *q*-value<0.05.

### Identification of differentially expressed proteins in PD patients

**Figure 5:**
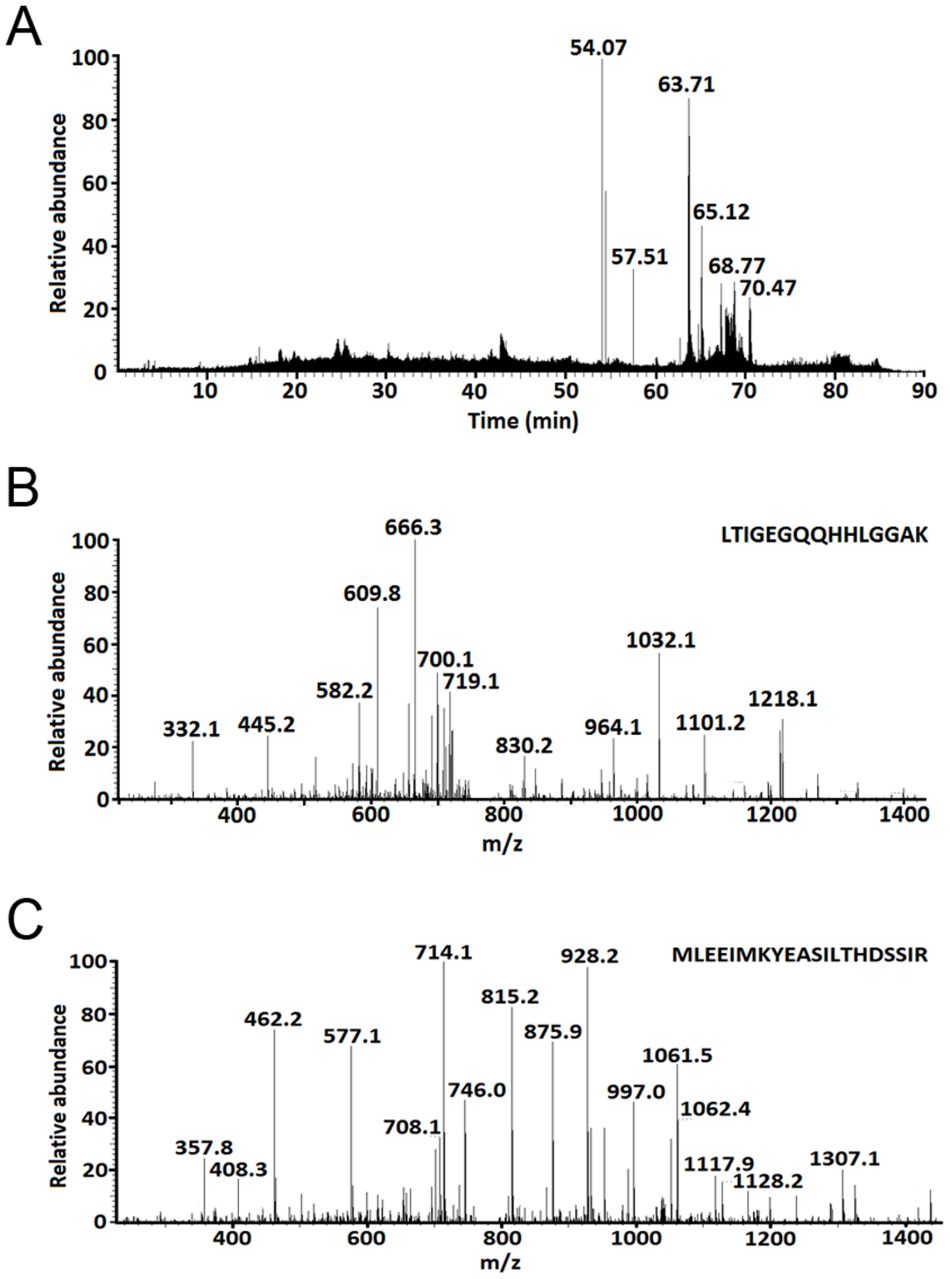
Representative results of the identification of protein (FGG) by LC-MS/MS. (A) Chromatogram of peptide mixture. (B and C) MS/MS spectra of two peptides.

To identify the differentially expressed proteins, the above-mentioned spots were excised for in-gel trypsin proteolysis and subsequently identified by LC-MS/MS analysis and the results are shown in Table 2 and Table 3. All these 13 differentially expressed protein spots corresponded to 6 different proteins, among which five proteins were represented by at least two distinct spots on the 2-DE gels. The 5 spots with increased expression in PD patients were identified as fibrinogen gamma chain (FGG) (spots 209, 213, 214, and 390) and haptoglobin (spot 14), and the 8 spots with decreased expression relative to the normal subjects were identified as prothrombin (spots 429 and 277), inter-alpha-trypsin inhibitor heavy chain H4 (ITI-H4) (spots 422 and 393), apolipoprotein A-I (spots 278 and 41), and apolipoprotein A-IV (Apo A-IV) (spots 268 and 266). The representative results of the LC-chromatogram and MS/MS spectra of two identified peptides from FGG were shown in Figure 5.

**Table 2:** Identification data of proteins significantly altered in the serum from PD patients compared with controls.

Spot No.^A^	Protein identity	UniProt Accession No.	Score	Sequence coverage (%)	p*I* B	MW^C^	PD/Control^D^
209	fibrinogen gamma chain	P02679	40.2	17.3	5.70	49496.5	10000
213	fibrinogen gamma chain	P02679	288.3	62.9	5.70	49496.5	10000
214	fibrinogen gamma chain	P02679	72.2	20.3	5.70	49496.5	9.5
390	fibrinogen gamma chain	P02679	240.3	59.3	5.70	49496.5	6.9
14	haptoglobin	P00738	88.2	20.9	6.13	45205	2.3
429	prothrombin	P00734	60.3	13	5.55	70036.5	−10000
277	prothrombin	P00734	70.3	17.7	5.55	70036.5	−97.8
422	inter-alpha-trypsin inhibitor heavy chain H4	Q14624	60.2	11.9	6.53	103357.7	−17.6
278	apolipoprotein A-I	P02647	90.2	33.3	5.46	30777.6	−4.2
268	apolipoprotein A-IV	P06727	100.3	26.3	5.16	45372.7	−4.1
393	inter-alpha-trypsin inhibitor heavy chain H4	Q14624	120.3	14.4	6.53	103357.7	−3.8
266	apolipoprotein A-IV	P06727	30.2	29	5.16	45372.7	−2.2
41	apolipoprotein A-I	P02647	150.3	44.2	5.46	30777.6	−2.1

Serum proteins from PD patients and controls were separated by 2-DE. Their identities were determined by LC-MS/MS as described in Materials and Methods.

A Spot numbers correspond to those shown in Figure 3.

B p*I* is the theoretical p*I* calculated from the amino acid sequence.

C MW refers to the theoretical molecular mass in kDa calculated from the amino acid sequence.

D Ratio PD/Control represents the ratio of spot intensity of the PD group to that of the same spot in the control group.

**Table 3:** Identified peptides for each differentially expressed protein (spot) analyzed by LC-MS/MS.

Spot No.^A^	Peptides identified
209	DNCCILDER, FGSYCPTTCGIADFLSTYQTK, AIQLTYNPDESSKPNMIDAATLK, KMLEEIMKYEASILTHDSSIR, MLEEIMKYEASILTHDSSIR, YEASILTHDSSIR, YLQEIYNSNNQK, VAQLEAQCQEPCKDTVQIHDITGKDCQDIANK, DTVQIHDITGK
213	DNCCILDER, FGSYCPTTCGIADFLSTYQTK, AIQLTYNPDESSKPNMIDAATLK, KMLEEIMKYEASILTHDSSIR, YLQEIYNSNNQK, VAQLEAQCQEPCKDTVQIHDITGKDCQDIANK, QSGLYFIKPLK, ANQQFLVYCEIDGSGNGWTVFQKR, LDGSVDFKK, EGFGHLSPTGTTEFWLGNEK, IHLISTQSAIPYALRVELEDWNGR, TSTADYAMFK, VGPEADKYR, CHAGHLNGVYYQGGTYSK, ASTPNGYDNGIIWATWK, LTIGEGQQHHLGGAK
214	FGSYCPTTCGIADFLSTYQTK, AIQLTYNPDESSKPNMIDAATLK, MLEEIMKYEASILTHDSSIR, VAQLEAQCQEPCKDTVQIHDITGK, VAQLEAQCQEPCKDTVQIHDITGKDCQDIANK, QSGLYFIKPLK, EGFGHLSPTGTTEFWLGNEK, IHLISTQSAIPYALRVELEDWNGR, ASTPNGYDNGIIWATWK
390	DNCCILDER, FGSYCPTTCGIADFLSTYQTK, AIQLTYNPDESSKPNMIDAATLK, KMLEEIMKYEASILTHDSSIR, YLQEIYNSNNQK, VAQLEAQCQEPCKDTVQIHDITGKDCQDIANK, QSGLYFIKPLK, ANQQFLVYCEIDGSGNGWTVFQKR, LDGSVDFKK, EGFGHLSPTGTTEFWLGNEK, IHLISTQSAIPYALRVELEDWNGR, TSTADYAMFK, VGPEADKYR, CHAGHLNGVYYQGGTYSK, ASTPNGYDNGIIWATWK, TRWYSMK, LTIGEGQQHHLGGAK
14	DIAPTLTLYVGK, DIAPTLTLYVGKK, VMPICLPSKDYAEVGR, YVMLPVADQDQCIR, SPVGVQPILNEHTFCAGMSK, SCAVAEYGVYVK, VTSIQDWVQK
429	SEGSSVNLSPPLEQCVPDR, SEGSSVNLSPPLEQCVPDRGQQYQGR, LAVTTHGLPCLAWASAQAK, ALSKHQDFNSAVQLVENFCR, HQDFNSAVQLVENFCR, NPDGDEEGVWCYVAGK
277	SEGSSVNLSPPLEQCVPDR, LAVTTHGLPCLAWASAQAK, HQDFNSAVQLVENFCR, NPDGDEEGVWCYVAGK, TATSEYQTFFNPR, TFGSGEADCGLRPLFEK, ELLESYIDGR
422	QGPVNLLSDPEQGVEVTGQYER, AGFSWIEVTFK, NPLVWVHASPEHVVVTR, WKETLFSVMPGLK, FSSHVGGTLGQFYQEVLWGSPAASDDGR, RLDYQEGPPGVEISCWSVEL
278	VKDLATVYVDVLK, DSGRDYVSQFEGSALGK, LLDNWDSVTSTFSK, LREQLGPVTQEFWDNLEK, EQLGPVTQEFWDNLEK, AKVQPYLDDFQK, KWQEEMELYR, THLAPYSDELR, QGLLPVLESFK, VSFLSALEEYTK
268	SELTQQLNALFQDK, LGEVNTYAGDLQKK, KLVPFATELHER, DSEKLKEEIGK, LKEEIGKELEELR, EEIGKELEELR, ENADSLQASLRPHADELK, IDQNVEELK, LNHQLEGLTFQMKK
393	QGPVNLLSDPEQGVEVTGQYER, AGFSWIEVTFK, NPLVWVHASPEHVVVTR, WKETLFSVMPGLK, TGLLLLSDPDK, VTIGLLFWDGR, FSSHVGGTLGQFYQEVLWGSPAASDDGRR, RLDYQEGPPGVEISCWSVEL
266	LGEVNTYAGDLQK, KLVPFATELHER, LKEEIGKELEELR, RQLTPYAQR, VLRENADSLQASLRPHADELK, KIDQNVEELKGR, VKIDQTVEELRR, SLAPYAQDTQEK, LNHQLEGLTFQMK, ISASAEELR
41	VKDLATVYVDVLKDSGR, DLATVYVDVLKDSGRDYVSQFEGSALGK, LLDNWDSVTSTFSK, LREQLGPVTQEFWDNLEK, ETEGLRQEMSKDLEEVK, QEMSKDLEEVKAK, VQPYLDDFQKK, WQEEMELYR, VEPLRAELQEGAR, QKLHELQEK, LSPLGEEMR, ARAHVDALR, THLAPYSDELRQR, LAARLEALKENGGAR, ATEHLSTLSEK, AKPALEDLRQGLLPVLESFK, VSFLSALEEYTKK

A Spot numbers correspond to those shown in Figure 3.

2-DE map allowed the high resolution of slight charge differences. FGG was observed as 4 distinct protein spots (spot 209, 213, 214, and 390) with almost the same MW (∼50 kDa) but slightly different p*I*, presumably representing protein modification. All these forms of the protein are up-regulated (spot 214 and 390) or freshly appeared (spot 209 and 213) in the serum from PD patients. Based on the present mass spectral data, the causes for this phenomenon of FGG in the serum of PD patients could not yet be ascertained.

### Western blot analysis using depleted serum

To confirm the disregulation in protein levels observed in the 2-DE analysis, Western blot analyses were carried out on FGG and ITI-H4, which *q*-values were smaller than 0.05, using the high abundant proteins depleted serum samples. Due to it was reported previously that Apo A-IV might participate in the pathology of neurodegenerative diseases such as Alzheimer's disease [18], [19], so we also verified the expression of Apo A-IV by Western blot analysis.

**Figure 6:**
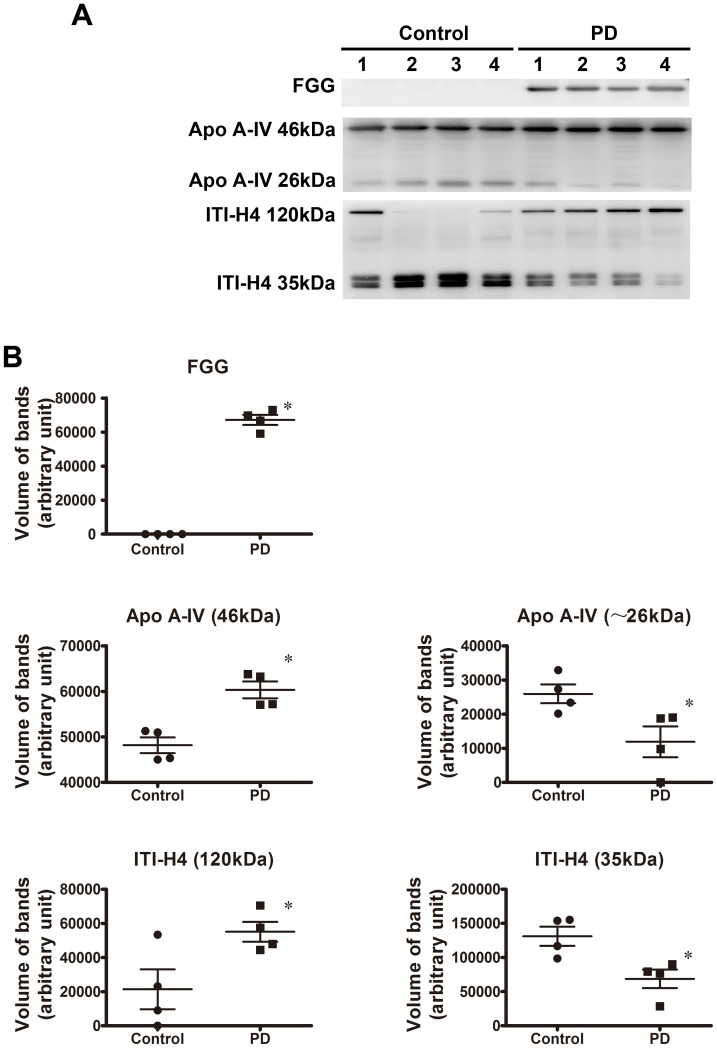
Expression of FGG, Apo A-IV, and ITI-H4 in individual depleted serum samples from 4 PD patients and 4 control subjects. (A) Representative panel of Western blots. It shows that full size (46 kDa) and ∼26 kDa fragment of Apo A-IV and full size (120 kDa) and 35 kDa fragment of ITI-H4 existed in PD and control serum. (B) Quantitative comparison of the Western blot shown in (A). FGG was only detected in PD serum samples. The level of full size Apo A-IV was increased, whereas fragmented Apo A-IV was decreased in PD patients compared with controls. The level of full size ITI-H4 was increased, whereas fragmented ITI-H4 was decreased in PD patients compared with controls. Total protein concentration in each sample was determined by Bradford assay. Protein loadings were approximately equal for all samples (20 µg/lane). Data represent mean ± S.E.M. for 4 individual subjects per group. *, *p*<0.05 compared with control, Student's *t*-test.

As shown in Figure 6, FGG (∼50 kDa) only expressed in PD serum samples, whereas there was no expression of FGG in control serum samples. Interestingly, the level of full size Apo A-IV (46 kDa) in PD serum samples was 1.3-fold higher than in control serum samples, however, the level of the fragmented Apo A-IV (∼26 kDa) was reduced by 37.3% in PD serum samples compared with control serum samples. Combine the localization of spot 266 and 268 in 2D gel (two spots appeared in approximate 30 kDa region of 2D map) and the results of Western blot analysis, spot 266 and 268 were the truncated protein (∼26 kDa) and not the full size protein (46 kDa).

Since the two spots identified as ITI-H4 (Spot 393 and 422) were appeared in approximate 35 kDa region of 2D map, therefore, Western blot analysis was performed to detect the fragmented bands of ITI-H4. It was found the band with molecular weight of 35 kDa, in addition to the bands of full size ITI-H4 (120 kDa). As shown in Figure 6, the level of fragmented ITI-H4 (35 kDa) was reduced by 47.6% in PD serum samples compared with control serum samples, whereas the level of full size ITI-H4 (120 kDa) in PD serum samples was 2.5-fold higher than in control samples. Hence, spot 393 and 422 were the truncated protein (35 kDa) and not the full size protein (120 kDa). These results were consistent with the data from 2D gel-based proteomic profiling in the present study.

### Validation of the protein expressions in whole serum

To determine whether the changes observed in depleted serum samples also occurred in whole serum samples, the levels of the most significantly disregulated proteins, FGG, Apo A-IV and ITI-H4 were also examined by western blotting of a set of whole serum samples from PD patients and controls.

**Figure 7:**
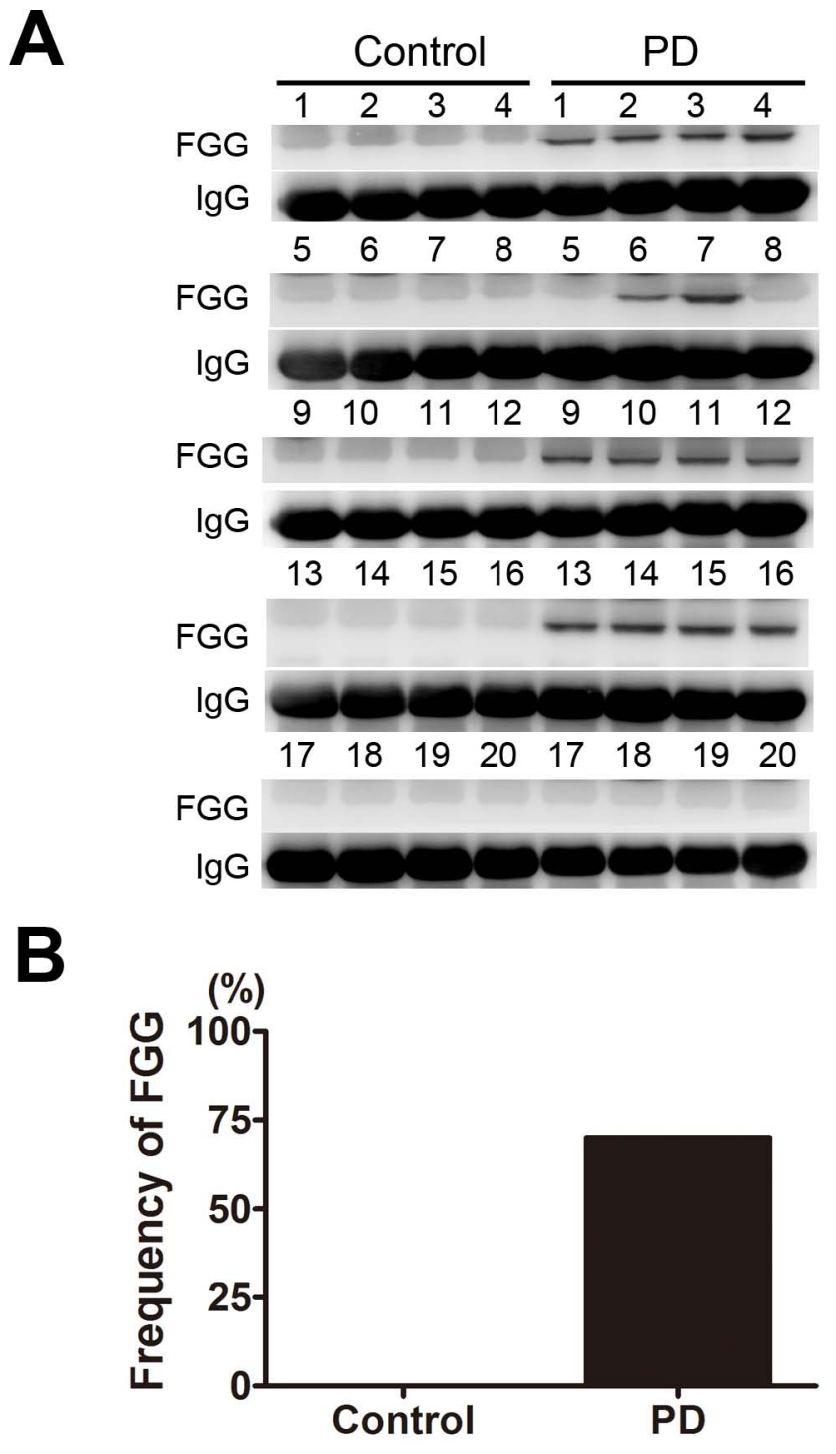
Validation of FGG in individual whole serum samples from 20 PD patients and 20 control subjects. (A) Representative panel of Western blots. (B) The frequency (14/20) of FGG detected in the serum of PD patients. None was detected in controls. Each number in the panels corresponds to an individual normal control or PD patient. Total protein concentration in each sample was determined by Bradford assay. Protein loadings were approximately equal for all samples (20 µg/lane). The immunoglobulin (IgG) was used as a loading control.

As shown in Figure 7, the expression of FGG was detected in 14 patients out of 20 PD patients, whereas there was no expression of FGG in 20 control serum samples (Figure 7A).

**Figure 8:**
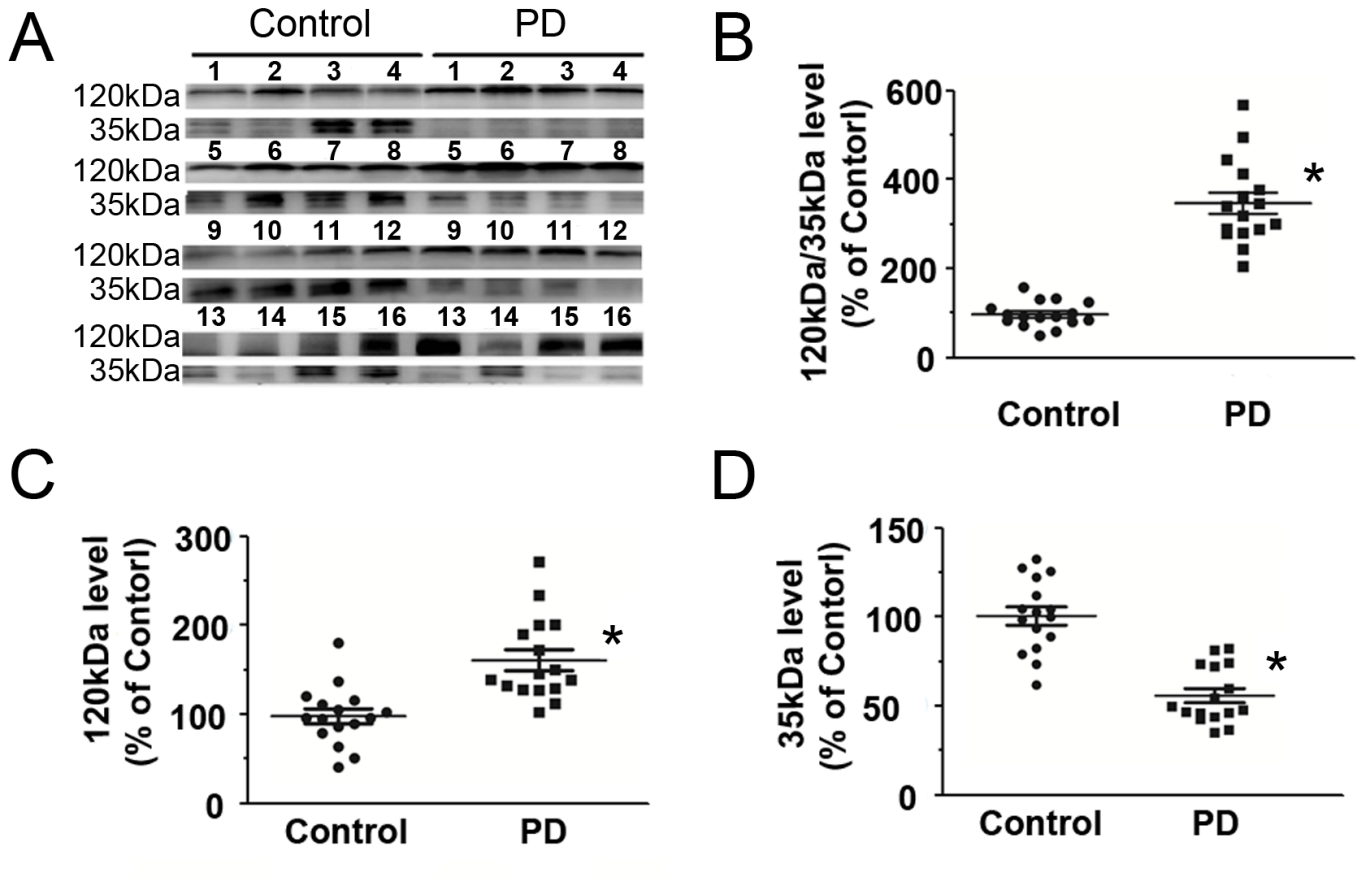
Expression of ITI-H4 in individual whole serum samples from 16 PD patients and 16 control subjects. (A) Representative panel of Western blots. Full size (120 kDa) and a fragment (35 kDa) of ITI-H4 were detected in the serum. (B) The ratio of full size to fragment ITI-H4 in PD serum was significantly higher than in control. (C and D) Quantitative analysis of the Western blot for full size and the fragmented ITI-H4, respectively, shown in (A). The level of full size ITI-H4 was increased, whereas fragmented ITI-H4 was decreased in PD patients compared with controls. Each number in the panels corresponds to an individual patient or control. Total protein concentration in each sample was determined by Bradford assay. Protein loadings were approximately equal for all samples (20 µg/lane). Data represent mean ± S.E.M. for 16 individual subjects per group. *, p<0.05 compared with control, Student's *t*-test.

The expression of ITI-H4 in whole serum samples from 16 PD patients and 16 controls was analyzed. Two bands were detected, one of them was full size ITI-H4 with a molecular weight of 120 kDa and another one was fragment of ITI-H4 with the molecular weight of 35 kDa (Figure 8A). It was interesting that the full size ITI-H4 (120 kDa) was increased in PD patients, whereas, fragmented ITI-H4 (35 kDa) was decreased in PD patients (Figure 8C, D). The both expressions of full size and fragmented ITI-H4 in control group were normalized to 1, the ratio between full size ITI-H4 (120 kDa) and fragmented ITI-H4 (35 kDa) in PD patients was 3.85±0.29-fold higher than in controls (1±0.27, p<0.05). The 95% confidence interval was between 0.86 and 1.14 in control group, as well as 3.23 and 4.47 in PD, respectively (Figure 8B). The difference between two groups was highly significant.

**Figure 9:**
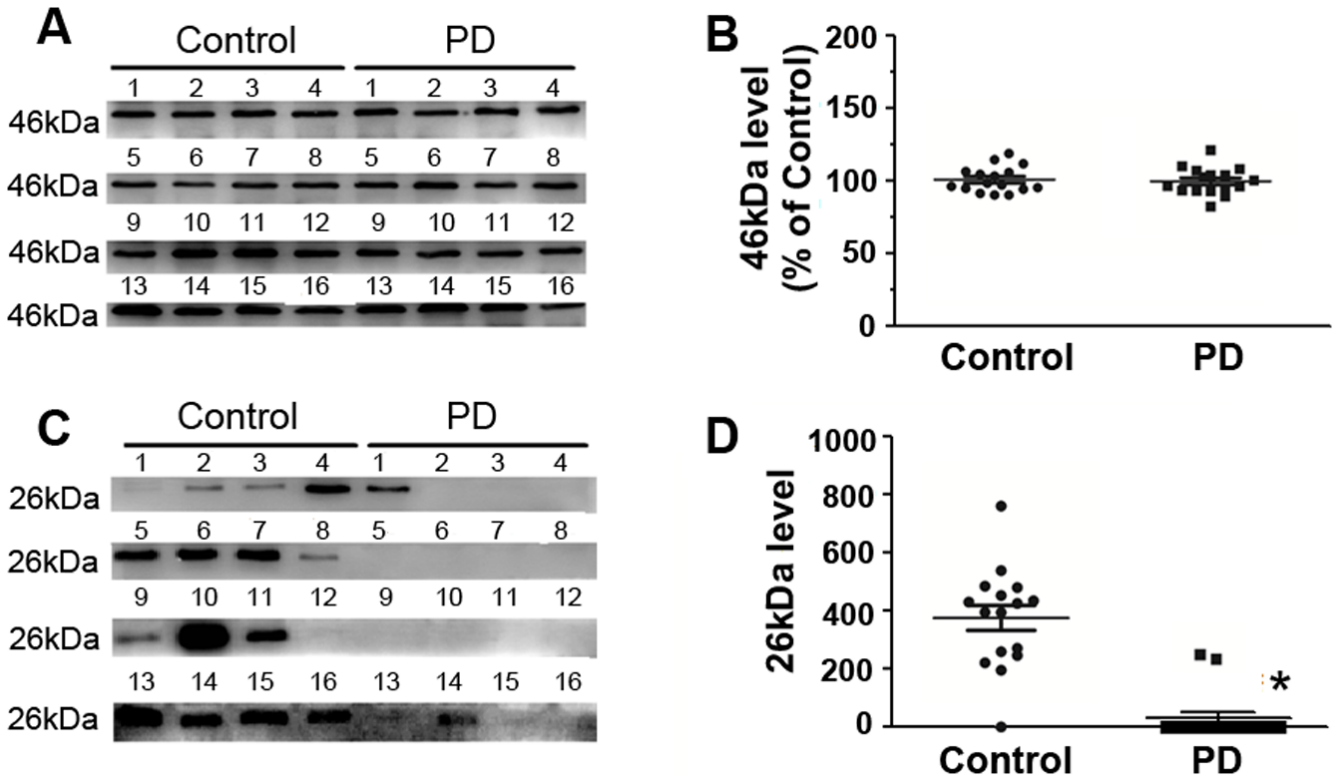
Expression of Apo A-IV in individual whole serum samples from 16 PD patients and 16 control subjects. (A and C) Representative panel of Western blots for full size (46 kDa) and the fragmented (∼26 kDa) Apo A-IV. (B and D) Quantitative analysis of the Western blot for full size and the fragmented Apo A-IV shown in A and C. The level of full size Apo A-IV was similar in control group and in PD patients. However, the fragmented Apo A-IV was mainly detected in control group. Each number in the panels corresponds to an individual patient or control. Total protein concentration in each sample was determined by Bradford assay. Protein loadings were approximately equal for all samples (20 µg/lane). Data represent mean ± S.E.M. for 16 individual subjects per group. *, *p*<0.05 compared with control, Student's *t*-test.

The expression of Apo A-IV in whole serum samples from 16 PD patients and 16 controls was also measured. It was found that there were also two bands in western bolt analysis, one with 46 kDa and another one with ∼26 kDa molecular weight (Figure 9A, C). The expression level of the protein at 46 kDa band was similar in control group and in PD group. However, the ∼26 kDa protein was mainly detected in control group. It was rarely found in PD group (Figure 9B, D).

These findings agree with our 2DE results and suggest that FGG, ITI-H4 and Apo A-IV were not only changed in expression quantity in the serum of PD patients, they were also altered the fragment or subunit constituents during PD.

## Discussion

Some potential protein biomarkers in the blood and cerebrospinal fluid have been pursued for the diagnosis and staging of PD. DJ-1 and α-synuclein, two proteins critically involved in PD pathogenesis, have been tested as potential disease biomarkers, but results have been inconsistent [20], [21]. CSF levels of α-synuclein show a decrease or no change between patients with PD and controls [22]–[24]. Several other potential protein biomarkers for PD are currently being investigated but the results have been highly variable and somewhat non-specific. There is a profound need for accurate and specific biomarkers to aid in the primary diagnosis of PD.

Proteomics tools can be broadly applied to the study of mechanisms in human disease, and have been frequently used for the discovery of biomarkers. Recently, various proteins that may be involved in the pathogenesis of PD were identified using proteomics technology [25]–[28]. In this study, we employed 2-DE and LC-MS/MS techniques to identify changes in protein expression in the serum of PD patients relative to non-PD controls. To gain ideal 2-DE protein profiles, we optimized the sample pretreatment to deplete the highly abundant serum proteins by MARS and established a 2-DE protocol. In total, we identified 13 protein spots, corresponding to 6 different gene products that were differentially expressed. Among them, 3 proteins changed in the level of protein fragment in PD patients confirmed by Western blot analysis. The failure of some of the LC-MS/MS identified proteins to be confirmed by western blotting may just reflect that the more sensitive techniques are required.

Among these proteins, the expression of FGG was found to be most altered in the PD samples. According to previous studies [10], [11], [13], [15], [29], [30], 5 of the proteins we observed as differentially expressed, namely fibrinogen gamma chain, haptoglobin, prothrombin, apolipoprotein A-I and apolipoprotein A-IV, have already been associated with PD. However the specific and the repeatable data were not satisfied with total protein changes. We report here for the first time that Apo A-IV and ITI-H4 were disregulated in the level of their specific fragments in the serum from PD patients.

Previous study demonstrated that high fibrinogen level in blood is associated with increased risk of PD among men over 75 years [31]. A recent study provided evidence that fibrinogen was different in CSF between PD and normal control populations [32]. It was known that fibrinogen is a blood-borne glycoprotein comprised of three pairs of non-identical polypeptide chains, eg. α, β and γ chains [33]. Fibrinogen γ chain increasing in PD serum was found and validated by 2DE and the western blot with specific antibody in the present study.

By means of lymphocyte proteomics of Parkinson's disease patients, it was demonstrated that two different isoforms of gamma-fibrinogen either correlate with the disease state or with the disease duration [10]. FGG is not produced by lymphocytes, but specific FGG isoforms are tightly bound by lymphocyte-specific receptors and regulate lymphocyte activities [34]. It is thus possible that the over expressed FGG in the serum might be dissociated from fibrinogen and it might be a pathological change in PD.

The functional features of the FGG include participation in fibrin polymerization and cross-linking, the initiation of fibrinolysis, a role in binding and regulating factor XIII activity, high affinity binding sites for integrin of platelets, leukocyte, and a role in mediating thrombin binding to fibrin, an inhibitory function originally termed ‘antithrombin I’ [35]. It appears that thrombotic and inflammatory mechanisms are probably both implicated in the effects of fibrinogen as this has been evaluated by several studies indicating that this acute phase glycoprotein is able to act as an inflammatory as well as a thrombotic marker [36]. It was suggested that inflammation may play a role in the neurodegenerative processes leading to PD [37]–[41].

In the present study, FGG was found to exist in four spots with a shift of p*I*, which might be due to the specific modification of this protein in PD. Furthermore, it was found that FGG only expressed in the PD samples. The significance of the change in FGG in serum of PD patients remains to be elucidated. The frequency of FGG expression was 70% in 20 PD patients, whereas there was no FGG detected in 20 normal controls. Although the number of the PD patients in this study was relatively low, the overexpression of FGG was confirmed in most of the patients. Therefore, we suggest that FGG may play a role in the pathogenesis of PD and may be used as a potential biomarker for PD.

Apo A-IV is a glycoprotein synthesized mainly in intestine and also in hypothalamus. The elevated level of Apo A-IV in CNS subsequently inhibits food intake and regulates the long-term balance of body weight [42], [43]. A recent study revealed that Apo A-IV showed a significant increase in serum of PD patients [15]. More recently a study suggested that Apo A-IV in plasma can be cleaved by matrix metalloproteinase (MMP)-7 into several fragments of 41, 32, 29, 27, 24, 22 and 19 kDa. The MMP-7-mediated cleavage of apo A-IV resulted in a rapid loss of its intrinsic anti-oxidant activity [44]. In the present study, we report that fragmented Apo A-IV (∼26 kDa) was almost removed in serum from PD patients for the first time, meanwhile, the levels of full size Apo A-IV (46 kDa) was not changed in PD serum validated by western blot, suggesting that the disregulation of the protein may play a role in the pathogenesis of PD. The effects of the altered Apo A-IV constituent on the function of the protein remain to be studied. Anyway, the fragmented Apo A-IV (∼26 kDa) might be a potential biomarker of PD.

The inter-α-trypsin inhibitor (ITI) family is a group of plasma proteins built up from heavy (HC1, HC2, HC3) and light chains synthesized in the liver. Although the function of ITI-H4 remains unclear, it has been shown that ITI-H4 is active in acute-phase inflammation [45], [46]. In previous studies, it has been indicated that ITI-H4 was apparently up-regulated in serum samples of patients with ovarian, breast or bladder cancers, and may provide important diagnostic information during surgical trauma [45], [47], [48]. Moreover, recent findings suggest that different cleaved forms of ITI-H4, such as 85 kDa ITI-H4 and 36 kDa ITI-H4, were disregulated in the serum in amyotrophic lateral sclerosis and recurrent pregnancy loss respectively [49], [50]. The changes of the fragments of ITI-H4 in PD patients were not reported. In the present study, we suggest that disregulation of the 35 kDa fragmented ITI-H4 and full size ITI-H4 might related to the PD pathogenesis, and the ratio between full size ITI-H4 (120 kDa) and fragmented ITI-H4 (35 kDa) in the serum may serve as a diagnostic indicator for PD.

Our results have showed that the FGG, Apo A-IV and ITI-H4 as well as their subunits might be potential biomarkers of PD. Due to easy detection of the subunits of above proteins from the serum of patients by using western blot analysis we suggest a combination use of these potential biomarkers for a high sensitivity and specificity diagnosis for PD. For example, it might be indicated to suffer from PD in the patients when FGG exists obviously or Apo A-IV ∼26 kDa fragment disappears in serum, or the ratio of ITI-H4 subunits 120 kDa and 35 kDa is larger than 3.20 (95% confidence interval for control group was 0.86 to 1.14 and for PD group was 3.23 to 4.47, respectively). If any two or all above three proteins are disregulated same times, might suggest to diagnosis the PD strongly. However, our suggestion is still needed to be verified by large scale clinical studies. In addition, in the present study all PD patients received dopamine agonist/L-Dopa treatment due to the reason of the medicinal ethics, therefore, it should be rid of the potential effects of drugs on protein expression in serum in future studies [51].

In conclusion, our present study identified six differentially expressed proteins, which include FGG, ITI-H4, Apo A-IV, haptoglobin, prothrombin and Apo A-I, in the serum of PD patients by using comparative proteomics. Especially FGG, ITI-H4 and Apo A-IV fragments, we suggest that might be used as powerful diagnostic biomarkers of PD by easily analyzing their levels in blood samples. Also, our results would provide information of drug targets for PD treatment.

## Materials and Methods

### Ethics Statement

This study was approved by the Institutional Review Board of Peking Union Medical College Hospital, Chinese Academy of Medical Sciences, and written informed consent was obtained from each participant.

### Human serum sample

Human serum specimens were collected from 21 sporadic PD patients (males with a mean age of 58.8±1.6) after diagnosis of PD for 1∼3 years and 20 normal individuals (males with a mean age of 60.8±1.6). The diagnosis of PD was based on clinical criteria previously described by Hughes *et al.* [52]. These PD patients showed Hoehn and Yahr Staging at 1.5–3.0 and appeared to have no family history of the disease. They all received sort of anti-parkinsonian medication, dopamine agonists and/or levodopa, and had good response. Patients with postencephalitic and drug-induced parkinsonism or a parkinson-plus syndrome were excluded. The normal individuals had no history, symptoms or signs of psychiatric or neuronal disease. All enrolled subjects signed informed consents. Human blood was obtained by venipuncture from each donor into evacuated blood collection tubes that contained no anticoagulant. The specimens were centrifuged at 4000 rpm for 15 min at 4°C. The resultant sera were stored at −80°C and transported on dry ice.

### Depletion of the highly abundant serum proteins by MARS

Serum samples were thawed, diluted with 5 vol of buffer A (Agilent Technologies, Product No. 5185-5987, pH 7.4) and centrifuged through a 0.22 mm filter membrane at 16,000 rpm. The prepared samples were maintained at 4°C and immediately used for further treatment. On an 1100 LC system (Agilent Technologies), each aliquot of the sample (equal to 20 mL original serum) was injected on a Multiple Affinity Removal System (MARS) HPLC column (Agilent Technologies, Palo Alto, CA, USA) for the depletion of the fourteen serum proteins of highest abundance according to the manufacturer. The flow-through fractions and the retained fractions were collected manually. All these fractions were stored at −80°C if not used immediately for further treatment.

### Desalting and concentrating the flow-through fractions by centrifugal ultrafiltration

The Centriplus centrifugal concentrators (YM-3, MWCO 3 kDa, Millipore) were rinsed and used according to the manufacturer's protocol. A 4 mL aliquot of the flow-through fraction was loaded each time. The sample was centrifuged at 4000 rpm, 4°C until the volume had decreased to about 150 µL. The concentrated solution was transferred into an Eppendorf vial. Protein content was determined by a 2-D Quant Kit (GE Healthcare).

### 2-DE analysis

Individual 2-DE gel of single sample from one subject was run. The first dimension was performed on Ettan IPGphor II IEF system (GE Healthcare). 350 µl rehydration buffer (8Murea, 2%CHAPS, 0.5% IPG buffer (pH 3–10 NL), 1% Destreak reagent (GE Healthcare), and 0.002% bromphenol blue) containing 120 g protein were loaded onto nonlinear IPG strips (18 cm, pH 3–10 NL, GE Healthcare). The isoelectric focusing was performed at 50 V for 12 h linearly; 200 V for 1 h linearly; 500 V for 1 h linearly, 1000 V for 1 h linearly; 8000 V for 1 h rapidly; and finally achieved 60,000 Vh at the voltage of 8000 V. The strips were then equilibrated for 15 min in equilibration buffer containing 6M urea, 0.05M Tris-Cl (pH 8.8), 2% sodium dodecyl sulfate (SDS), 30% glycerol, and 1% DTT, and re-equilibrated for 15 min in the same buffer containing 2.5% iodoacetamide in place of DTT. The equilibrated gel strip was placed on the top of a 12.5% SDS-polyacrylamide gel electrophoresis (PAGE) gel, and embed with 0.5% low-melt agarose (Sigma). Then the second dimension separation was performed in an Ettan DALTsix Electrophoresis System (GE Healthcare) at 16°C as follows: 2.5 W/gel constant powers for 45 min, and then 17 W/gel constant powers until the bromophenol blue front reached the bottom of the gels.

### Gel staining and image analysis

After performing 2-DE, the analytic gels were silver stained using PlusOne Silver Staining Kit (GE Healthcare). The silver stained gels were scanned using ImageScanner III scanner (GE Healthcare). Image analysis was carried out by using ImageMaster 2D Platinum software 7.0 (GE Healthcare). Concretely, Protein spots in the gels were detected automatically by the software and manually refined, and each spot was quantified on the basis of its normalized volume (spot volume normalized to the sum of the volumes of all the representative spots in a gel) to prevent variation between different 2-DE gels. Gels were then automatically matched by the software and the resulting clusters of spots confirmed manually.

The normalized volume of each spot from forty-one individual gels from the two cohorts was compared between different groups using Student's *t*-test provided by ImageMaster 2D Platinum software 7.0. Meanwhile, variation ratio of protein spot volumes between two different groups was calculated by the software. The variation ratio was the maximum ratio between the lower limit of the group with higher mean value and the upper limit of the other group. Absolute values smaller than 1 indicate overlap, whereas absolute values higher than 1 show that there is no overlap between intervals of two groups. In order to easily distinguish matched spots that are over or under expressed in one of the groups, “Ratio = 10000” represented the characteristic that the spot did not exist in one cohort, that was the difference of “all” or “none”, “+” and “−” represent “up-regulation” and “down-regulation”, respectively. Only spots with *P*-values<0.05 and the ratio >2 were considered as differentially expressed protein spots. Checked the results by visual inspection of the differentially expressed protein spots to avoid the erroneous conclusions due to inaccuracies in detection or matching. To solve the problem of multiple hypotheses testing, *q*-values, representing the FDR adjusted *P*-values, were evaluated for each matched spot using an Excel spreadsheet developed by Dr Graham Horgan of Biomathematics and Statistics Scotland (downloaded from http://www.rowett.ac.uk/~gwh/qval.xls) and based on the equations of Storey and Tibshirani [53].

### Identification of candidate protein biomarkers by LC-MS/MS

Above differentially expressed protein spots were manually excised from the silver stained gels. In-gel digestion of each protein spot was performed with a standard protocol. Briefly, each protein spot was dissolved in 25 mM NH_4_HCO_3_, reduced with 10 mM DTT for 1 hour at 56°C, and alkylated by 40 mM iodoacetamide in the dark for 45 minutes at room temperature. Then, protein spots were digested using sequence-grade modified trypsin (Promega Corporation, USA) overnight at 37°C. LC-MS/MS analysis was performed using a ThermoFinnigan LTQ linear ion trap mass spectrometer in line with a ThermoFinnigan Surveyor MS Pump Plus HPLC system. Tryptic peptides generated above were loaded onto a trap column (300SB-C18, 5×0.3 mm, 5 µm particle) (Agilent Technologies, Santa Clara CA) which was connected through a zero dead volume union to the self-packed analytical column (C18, 100 µm I.D.×100 mm, 3 µm particle) (SunChrom, Germany). The peptides were then eluted over a gradient (0–45% B in 55 minutes, 45–100% B in 10 minutes, where B = 80% acetonitrile/0.1% formic acid) at a flow rate of 500 nL/min and introduced online into the linear ion trap mass spectrometer (ThermoFisher Corporation, San Jose, CA) using nanoelectrospray ionization (ESI). Data dependent scanning was incorporated to select the 5 most abundant ions (one microscan per spectra; precursor isolation width 1.0 m/z, 35% collision energy, 30 ms ion activation, exclusion duration: 90 s; repeat count: 1) from a full-scan mass spectrum for fragmentation by collision induced dissociation (CID). MS data were analyzed using SEQUEST against NCBI Reference Sequence human protein database and results were filtered, sorted, and displayed using the Bioworks 3.31. Peptides with +1, +2, or +3 charge states were accepted if they were fully enzymatic and had a cross correlation (Xcorr) of >1.80, >2.2, and >3.0, respectively.

### Western blot analysis

Human serum samples were subjected to SDS-PAGE to validate the data of proteomic analysis. Protein concentrations of each sample were determined using Bradford assay. Equivalent amounts of protein were loaded in each lane of a 12% SDS-PAGE and blotted onto nitrocellulose membranes. The membranes were incubated with blocking solution, containing a 1∶500 dilution of anti-fibrinogen gamma chain (Santa Cruz Biotechnology, Santa Cruz, CA, USA), anti-Apo A-IV (Santa Cruz Biotechnology, Santa Cruz, CA, USA), or anti-ITI-H4 antibodies (Santa Cruz Biotechnology, Santa Cruz, CA, USA). The membranes were then incubated with blocking solution containing a 1∶10000 dilution of rabbit anti-goat IgG-HRP or goat anti-mouse IgG-HRP as a secondary antibody. An ECL system (Las-3000 Luminescent Image Analyzer, Fujifilm) was used to detect signals from the Western blot. The quantitative analysis of the Western blot was carried out using the QuantityOne software (Bio-Rad).

### Statistical analysis

The normalized volume of each spot from forty-one individual gels was compared between different groups using Student's *t*-test provided by ImageMaster 2D Platinum software 7.0, and *P*-values were adjusted by FDR as *q*-values.

SPSS 13.0 software was used for statistical analyses in Western blot analysis. Data are expressed as means±SEM. Groups were compared by Student's *t*-test. Differences were considered significant at P<0.05.
